# Clinical massage therapy for patients with cancer-related fatigue protocol of a systematic review

**DOI:** 10.1097/MD.0000000000013440

**Published:** 2018-12-10

**Authors:** Kang Wang, Shuo Qi, Hezheng Lai, Xiaoshu Zhu, Guobing Fu

**Affiliations:** a Dongfang Hospital of Beijing University of Chinese Medicine; b Dongzhimen Hospital of Beijing University of Chinese Medicine, Beijing, China; c School of Science and Health, Western Sydney University, Campbelltown, New South Wales; d The Chinese Medicine Center, Collaboration Between Beijing University of Chinese Medicine and Western Sydney University, Australia.

**Keywords:** cancer-related fatigue, complementary medicine, massage, quality of life

## Abstract

**Background:**

Cancer-related fatigue (CRF) is a prevalent and debilitating symptom experienced by cancer survivors, one that severely compromises functional independence and quality of life. Clinical massage therapy (CMT), as an important part of complementary and alternative medicine, is widely employed among massage therapists, physical therapists, nurses, and physicians when managing CRF. Clinical research indicates that CMT produced relief of CRF. In this systematic review, we aim to evaluate the effectiveness and safety of CMT for patients with CRF.

**Methods:**

We will search the following electronic databases for randomized controlled trials to evaluate the effectiveness and safety of CMT for CRF in cancer patients: CENTRAL, Embase, MEDILINE, CINAHL and China National Knowledge Infrastructure. Each database will be searched from inception to October 2018. The entire process will include study selection, data extraction, risk of bias assessment and meta-analyses.

**Results:**

This proposed study will evaluate the effectiveness and safety of CMT for CRF. The outcomes will include change in quality of life, fatigue relief and adverse effect.

**Conclusions:**

This proposed systematic review will evaluate the existing evidence on the effectiveness and safety of CMT for patients with CRF.

**Dissemination and ethics:**

The results of this review will be disseminated through peer-reviewed publication. Because all of the data used in this systematic review and meta-analysis has been published, this review does not require ethical approval. Furthermore, all data will be analyzed anonymously during the review process.

## 1 Introduction

With approximately 15.5 million cancer survivors today in the United States alone, increased attention is being given to quality of life after cancer treatment.^[1]^ Cancer-related fatigue (CRF) is one of the most prevalent and debilitating symptoms experienced by people with cancer.^[2]^ It can persist for months or years after cancer therapy is completed^[3–5]^ and has a negative impact on all areas of function.^[6]^ Moreover, CRF is more troublesome and has a greater negative impact on quality of life than cancer-related pain, depression, or nausea.^[7,8]^

It is reported that over 50% of cancer survivors use a complementary and alternative medicine (CAM) approach for symptom management,^[9]^ and demand and expectation from the survivors keep increasing.^[10]^ One of the widely employed CAM interventions is massage therapy. Most studies that investigate massage therapy as supportive care for patients treated for cancer focus on depression, anxiety, or pain as the outcomes of interest.^[11–13]^

Nowadays, clinical massage therapy (CMT) is one of the fastest growing alternative therapies and has a high rate of acceptance for symptom management among cancer patients. There are many different styles of CMT, such as, Chinese massage, Swedish massage, Japanese massage, Thai massage, etc. All of which involve manipulating muscles and rubbing or stroking soft tissues of the body. Moreover, massage has been shown in smaller studies with cancer patients to modulate the immune system,^[14,15]^ and has been demonstrated to significantly decrease markers of immune system activation in normal subjects.^[16,17]^

Over the years, there has been a general concern that massage can increase the risk of cancer cells spreading to other parts of the body.^[18–20]^ However, there is lack of evidence that this happens. Only through some use of extreme focused pressure, such as sentinel lymph node mapping, that the promotion of metastasis through physical touch has been demonstrated.^[21]^

This review aims to systematically review all randomized controlled trials (RCTs) to assess the effectiveness and safety of CMT for patients with CRF.

## 2 Materials and methods

This systematic review protocol has been registered on PROSPERO. The Protocol follows the Cochrane Handbook for Systematic Reviews of Interventions and the Preferred Reporting Items for Systematic Reviews and Meta-Analysis Protocol (PRISMA-P) statement guidelines.^[22]^ We will describe the changes in our full review if needed.

## 3 Inclusion criteria for study selection

### 3.1 Type of studies

This review will include clinical RCTs of CMT for CRF in cancer patients without any language or publication status restrictions. Non-RCTs, quasi-RCTs, case series, case reports, crossover studies, uncontrolled trials, and laboratory studies will not be included.

### 3.2 Type of participants

Cancer patients, male or female, of any age with a diagnosis of CRF, which based on International Statistical Classification of Diseases and Related Health Problems, 10^th^ revision (ICD-10) proposed criteria and required evidence from the history, physical examination, and laboratory findings that the fatigue was a consequence of cancer or cancer therapy and not primarily a consequence of comorbid physical or psychiatric disorders, will be included. Pregnant and lactating women will be excluded.

Patients who were actively suicidal or homicidal; medical conditions felt to be clinically contributing to fatigue; medications felt to be clinically contributing to fatigue; body mass index<18.5 kg/m^2^; and use of systemic corticosteroids or other immunosuppressants within the past 6 months will be excluded.

### 3.3 Type of interventions

Interventions will include any type of clinically performed massage for improvement of cancer-related fatigue. This will include Chinese Massage, Japanese Massage, Thai Massage, Swedish Massage, Tuina, Shiatsu, Remedial Massage, General Massage, Acupressure, Reflexology, Manual Lymphatic Drainage. Studies of CMT combined with other interventions such as acupuncture, herbal medicines, qigong and yoga will be considered for exclusion.

Control: Placebo, no intervention, standard care and other body-based practices including exercise techniques, qigong, yoga and taichi.

### 3.4 Type of outcome measures

#### 3.4.1 Primary outcomes

The primary outcome measure will be Quality of Life Questionnaire Core 30 (QLQ-C30) from European Organization for Research on Treatment of Cancer (EORTC)

#### 3.4.2 Secondary outcome

The secondary outcome measures will include

Multidimensional Fatigue Inventory (MFI);

Patient Reported Outcomes Measurement Information System (PROMIS) Fatigue Short Form;

Quality of Life Enjoyment and Satisfaction Questionnaire (Q-LES-Q).

## 4 Search methods for the identification of studies

### 4.1 Electronic searches

We will search the following electronic bibliographic databases for relevant trials:

- Cochrane Central Register of Controlled Trials (CENTRAL);
- CINAHL (Cumulative Index of Nursing and Allied Health Literature, from 1937 to present);
- EMBASE (Excerpta Medica database, from 1947 to present);
- Ovid MEDLINE ALL (Ovid Medical Literature Analysis and Retrieval System Online, from 1946 to present);
- CNKI (China National Knowledge Infrastructure Database, from 1979 to present).

### 4.2 Data collection and analysis

#### 4.2.1 Study identification

We will use EndNote X8 software to manage the records of searched electronic databases. The initial selection will involve scanning of the titles and abstracts of the retrieved studies. The full text of relevant studies will then be reviewed for study inclusion, in accordance with the inclusion criteria, by 2 authors (KW and SQ). Potentially relevant articles will be reviewed independently by 2 authors (KW and HL) to determine if they meet the prespecified criteria. Any disagreement between authors will be resolved by consensus with a third author. The study selection procedure will follow and be recorded in the PRISMA flow chart. All the evidence will be assessed by The Grading of Recommendations Assessment, Development and Evaluation (GRADE).

#### 4.2.2 Data extraction and management

According to the inclusion criteria, a standard data collection form will be made before data extraction. The following data will be extracted by 2 authors (KW and SQ):

- *General information*: Research identification, publication year, the title of the study, first author.
- *Study methods*: Study design, sample size, randomization method, allocation. concealment, blinding, incomplete report or selecting report, other sources of bias;
- *Participants*: Inclusion and exclusion criteria.
- *Intervention*: CMT technique names, motion details, treatment duration, and frequency.
- *Control*: Type of control body-based practice, motion details, treatment duration, and frequency.
- *Outcomes*: Primary, secondary, and safety outcomes.

#### 4.2.3 Risk of bias assessment

The risk of bias in included studies will be assessed independently by 2 reviewers (KW and GF) using the Cochrane Risk of Bias Tool, with any disagreements resolved by consensus or by discussion with a third reviewer. All judgments will be fully described, and the conclusions will be presented in the Risk of Bias figures and will be incorporated into the interpretation of review findings, by means of sensitivity analysis. The risk of bias of each domain will be graded as adequate, unclear, or inadequate. We intend to use the concealment of allocation grading in investigation of any heterogeneity and in sensitivity analysis. Other aspects of study quality including the extent of blinding (if appropriate), the extent of losses to follow up, non-compliance, whether the outcome assessment was standardized, and whether an intention to treat analysis was undertaken, will be presented in the risk of bias table describing the included studies and will provide a context for discussing the reliability of the results.

#### 4.2.4 Data analysis

We will use Review Manager Software (Review Manager—RevMan) [Computer program]. Version 5.3. Copenhagen: The Nordic Cochrane Centre, The Cochrane Collaboration, 2014) provided by Cochrane Collaboration for data synthesis and analysis by 2 authors (XZ and HL). We will summarize data using risk radio (RR) with 95% confidence intervals (CI) for binary outcomes. If different measurement scales are used, standardized mean difference analyses will be performed. We will consider heterogeneity to be substantial if the *I*^2^ statistic is > 50%. Data from individual trials will be combined for meta-analysis if the interventions, patient groups and outcomes are sufficiently similar. Meta-analysis will not be performed if the *I*^2^ statistic is ≥85%. We will use a random-effects model for meta-analysis unless the degree of heterogeneity is readily explainable. If the *I*^2^ statistic is <25% a fixed-effect model will be used.

*Sensitivity analyses*: heterogeneity may be due to the presence of 1 or more outlier studies with results that conflict with the rest of the studies. We will perform sensitivity analyses excluding outlier studies. In addition, we plan to perform sensitivity analysis to explore the influence of trial quality on effect estimates. The quality components of methodology include adequacy of generation of allocation sequence, concealment of allocation, and the use of intention-to-treat analysis.

*Subgroup analyses*: if data permits, we will perform the following subgroup analyses.

- different types of outcome measures;
- different types of control therapies;
- treatment duration.

#### 4.2.5 Publication bias

If sufficient number of trials (more than 10 trials) are found, we will generate funnel plots (effect size against standard error) to investigate publication bias.

#### 4.2.6 Ethics and dissemination

The data used in this systematic review will be collected from published studies. Based on this, the study does not require ethical approval.

## Acknowledgment

The authors would like to thank all the researchers in our working group.

Appendix A. CENTRAL Search Strategy, https://www.crd.york.ac.uk/PROSPEROFILES/111739_STRATEGY_20181009.pdf

## Author contributions

KW, GF contributed on methodology and are the guarantors of the review.

KW, SQ, HL, XZ, and GF contributed on data search, analysis, and statistics.

HL contributed on the language editing.

Methodology: Kang Wang, Xiaoshu Zhu.

Resources: Guobing Fu, Hezheng Lai.

**Data curation:** Kang Wang, Shuo Qi.

**Funding acquisition:** Guobing Fu.

**Methodology:** Kang Wang.

**Project administration:** Kang Wang.

**Software:** Shuo Qi.

**Supervision:** Guobing Fu.

**Writing – original draft:** Kang Wang.

**Writing – review & editing:** Kang Wang, Hezheng Lai, Xiaoshu Zhu.

Kang Wang: 0000-0002-8894-5601

## References

[1] Kinkead B Schettler PJ Larson ER Massage therapy decreases cancer-related fatigue: results from a randomized early phase trial. Cancer 2018;124:546–54. 29044466 10.1002/cncr.31064 PMC5780237

[2] Kapoor A Singhal MK Bagri PK Cancer related fatigue: a ubiquitous problem yet so under reported, under recognized and under treated. South Asian J Cancer 2015;4:21–3. 25839015 10.4103/2278-330X.149942 PMC4382777

[3] Bower JE Ganz PA Desmond KA Fatigue in breast cancer survivors: occurrence, correlates, and impact on quality of life. J Clin Oncol 2000;18:743–53. 10673515 10.1200/JCO.2000.18.4.743

[4] Curran SL Beacham AO Andrykowski MA Ecological momentary assessment of fatigue following breast cancer treatment. J Behav Med 2004;27:425–44. 15675633 10.1023/b:jobm.0000047608.03692.0c

[5] Hofman M Ryan JL Figueroa-Moseley CD Cancer-related fatigue: the scale of the problem. Oncologist 2007;12 suppl 1:4–10. 17573451 10.1634/theoncologist.12-S1-4

[6] Jedlicka F Elbl L Vasova I Chronic fatigue syndrome in cancer patients. Diagnostic and treatment options. Vnitr Lek 2007;53:979–85. 18019669

[7] Curt GA Breitbart W Cella D Impact of cancer-related fatigue on the lives of patients: new findings from the Fatigue Coalition. Oncologist 2000;5:353–60. 11040270 10.1634/theoncologist.5-5-353

[8] Stone P Richards M A’Hern R A study to investigate the prevalence, severity and correlates of fatigue among patients with cancer in comparison with a control group of volunteers without cancer. Ann Oncol 2000;11:561–7. 10907949 10.1023/a:1008331230608

[9] Vapiwala N Mick R Hampshire MK Patient initiation of complementary and alternative medical therapies (CAM) following cancer diagnosis. Cancer J 2006;12:467–74. 17207316 10.1097/00130404-200611000-00006

[10] Hunter J Ussher J Parton C Australian integrative oncology services: a mixed-method study exploring the views of cancer survivors. BMC Complement Altern Med 2018;18:153. 29743054 10.1186/s12906-018-2209-6 PMC5944107

[11] Boyd C Crawford C Paat CF The impact of massage therapy on function in pain populations—a systematic review and meta-analysis of randomized controlled trials: part II, cancer pain populations. Pain Med 2016;17:1553–68. 27165967 10.1093/pm/pnw100 PMC4975018

[12] Greenlee H DuPont-Reyes MJ Balneaves LG Clinical practice guidelines on the evidence-based use of integrative therapies during and after breast cancer treatment. CA Cancer J Clin 2017;67:194–232. 28436999 10.3322/caac.21397 PMC5892208

[13] Mao JJ Wagner KE Seluzicki CM Integrating oncology massage into chemoinfusion suites: a program evaluation. J Oncol Pract 2017;13:e207–16. 28045616 10.1200/JOP.2016.015081 PMC5702787

[14] Billhult A Lindholm C Gunnarsson R The effect of massage on immune function and stress in women with breast cancer--a randomized controlled trial. Auton Neurosci 2009;150:111–5. 19376750 10.1016/j.autneu.2009.03.010

[15] Fernandez-Lao C Cantarero-Villanueva I Diaz-Rodriguez L The influence of patient attitude toward massage on pressure pain sensitivity and immune system after application of myofascial release in breast cancer survivors: a randomized, controlled crossover study. J Manipulative Physiol Ther 2012;35:94–100. 22018755 10.1016/j.jmpt.2011.09.011

[16] Carter CS Pournajafi-Nazarloo H Kramer KM Oxytocin: behavioral associations and potential as a salivary biomarker. Ann N Y Acad Sci 2007;1098:312–22. 17435137 10.1196/annals.1384.006

[17] Noto Y Kudo M Hirota K Back massage therapy promotes psychological relaxation and an increase in salivary chromogranin A release. J Anesth 2010;24:955–8. 20683736 10.1007/s00540-010-1001-7

[18] MacDonald G Massage therapy in cancer care: an overview of the past, present, and future. Altern Ther Health Med 2014;20 suppl 2:12–5. 25362212

[19] Chapman C Kennedy E Mastectomy massage. Massage Ther 2000;39:90–100.

[20] Curties D Could massage therapy promote cancer metastasis? Massage Ther 2000;39:83–90.

[21] Bass SS Cox CE Salud CJ The effects of postinjection massage on the sensitivity of lymphatic mapping in breast cancer. J Am Coll Surg 2001;192:9–16. 11192930 10.1016/s1072-7515(00)00771-7

[22] Shamseer L Moher D Clarke M Preferred reporting items for systematic review and meta-analysis protocols (PRISMA-P) 2015: elaboration and explanation. BMJ 2015;350:g7647. 25555855 10.1136/bmj.g7647

